# Characterization of a high-brightness, laser-cooled Li^+^ ion source

**DOI:** 10.1063/1.5085068

**Published:** 2019

**Authors:** J. R. Gardner, W. R. McGehee, J. J. McClelland

**Affiliations:** Nanoscale Device Characterization Division, National Institute of Standards and Technology, Gaithersburg, Maryland 20899, USA

## Abstract

Ion sources based on laser cooling have recently provided new pathways to high-resolution microscopy, ion milling, and ion implantation. Here, we present the design and detailed characterization of a ^7^Li magneto-optical trap ion source (MOTIS) with a peak brightness of (1.2 ± 0.2) × 10^5^ A m^−2^ sr^−1^ eV^−1^ and a maximum continuous current over 1 nA. These values significantly surpass previous Li MOTIS performance benchmarks. Using simple models, we discuss how the performance of this system relates to fundamental operating limits. This source will support a range of projects using lithium ion beams for surface microscopy and nanostructure characterization, including Li^+^ implantation for studies of ionic transport in energy storage materials.

## I. INTRODUCTION

Focused ion beams (FIBs) are the basis for an expanding list of nanoscale manufacturing and characterization techniques. They serve as versatile nanofabrication tools,^1-8^ powerful microscopes,^9–11^ and precise ion implantation devices.^12-16^ In all of these capacities, a FIB’s utility is dependent on how well it focuses on the nanoscale—a property fundamentally limited by the brightness and the energy spread of the ion source. Ion sources based on cold atoms have recently demonstrated focusing competitive with, or even surpassing, more conventional liquid metal (LMIS) and gas field ionization sources (GFIS).^17–20^ Moreover, cold atom sources are robust under mechanical vibration, have inherent isotopic purity, and can be pulsed or ramped at nanosecond timescales. They are also, at least in principle, capable of producing ion beams consisting of any of the over 27 elements amenable to laser cooling—many of which are not accessible by conventional means.^1^

Cold atom ion sources generally consist of a gaseous source of neutral atoms, an array of laser beams tuned near a resonance in the atom, and one or more photoionization laser beams. The near-resonant laser light cools the neutral atoms to an extremely low temperature, typically in the microkelvin range, after which the atoms are photoionized and extracted electrostatically. Provided the ionization is done near threshold and Coulomb interactions are not too strong, the low temperature of the atoms is retained by the ions during and after extraction. The result is an ion beam with an extremely small transverse velocity spread and a correspondingly high degree of collimation. These beams are typically extracted from micrometer-scale ionization regions, the geometry of which is determined by the laser fields used for ionization.

The utility of a FIB ion-optical system depends on its ability to deliver a large number of ions into a small spot. An important figure of merit describing this characteristic is the normalized brightness, defined as the current density per solid angle per beam energy. For a thermal source with current density J(*x*, *y*) and temperature T, the normalized brightness is

(1) $$B(x,y)=\frac{J(x,y)}{\pi k_{B}T},$$

where *k*_B_ is Boltzmann’s constant. Related to the brightness is the emittance, $\varepsilon =\sigma \sqrt{k_{B}T∕2}$, where *σ* is the RMS deviation of transverse particle positions in the source. Emittance characterizes a source’s capacity for focusing to a small spot, with small emittance corresponding to better focusing. Brightness, by contrast, reflects the amount of current a beam can deliver to a small spot, with large brightness corresponding to large maximum current density. For a Gaussian source with uniform angular distribution, the peak normalized brightness can be written in terms of the emittance as B_p_ = I_0_/(4*π*^2^*ε*^2^), where I_0_ is the total current. The peak brightness of a beam is unchanged by propagation, energy changes, and (idealized) ion optics and provides a context-free metric for predicting how a source will behave in a given FIB column.

Conventional tip-based ion sources achieve small emittance by making *σ* extremely small, often in the few-nanometer regime—or even at the single-atom level in the case of the GFIS. In contrast, cold-atom-based sources rely on their extremely low source temperature to reach a suitably small emittance, even though *σ* is relatively large. The fact that both approaches can lead to sufficiently small emittance for high resolution focusing has an analogy in classical optics, where a light beam’s collimation and size can be interchanged without diminishing its capacity for focusing to a small spot.

Continuous innovation in cold-atom ion source design has yielded brightness on the order of 10^7^ A m^−2^ sr^−1^ eV^−1^ and sub-eV energy spreads.^18^ In particular, sources using only transverse cooling have been demonstrated with great success in cesium^17,18,21^ and rubidium.^22,23^ These species benefit from efficient sub-Doppler cooling and can be formed into bright atomic beams. Basic laser cooling of lithium is less efficient, limiting the utility of transverse-only techniques. As a compromise between experimental complexity and source brightness, we use a MOTIS design for the work described here. Slow transport rates within the magneto-optical trap (MOT) fundamentally limit ion current and brightness and are discussed in detail below.

Despite laser cooling limitations, lithium has significant value as a cold-atom FIB species because of its diagnostic potential and its behavior as an implanted ion.^1,24^ Due to its light mass, Li^+^ inflicts minimal sample damage—particularly at low energy (0.5 keV to 5 keV), where tip-based FIBs perform poorly. Compared with He^+^, Li^+^ ions have a low probability of recombination with target electrons, leading to higher ion backscatter yield.^25^ These properties make Li^+^ a promising species for surface microscopy. As an implanted ion, lithium plays a central role in certain electrochemical devices—most notably in rechargeable Li-ion batteries—where its nanoscale interactions and dynamics are incompletely understood. Low energy lithium FIBs provide a powerful tool for titrating controlled doses of Li^+^ into a sample, where its chemical and physical behavior provides rare insight into device performance.^12^

The first example of a cold-atom lithium FIB, described in Ref. 24, has demonstrated the utility of Li^+^ for FIB microscopy, surface characterization, and implantation.^10,12,26,27^ This source has a brightness on the order of 6000 Am^−2^ sr^−1^ eV^−1^, yielding a focused spot size (25% to 75% rise distance) of ≈ 27 nm at 2 keV beam energy and 1 pA current. The maximum continuous current obtained with this source is ≈ 60 pA. This performance is modest compared to the leading FIBs (both cold-atom and tip-based) and does not approach the theoretical limit attainable in a Li source.^1^ Nevertheless, ongoing research based on this system shows that low-energy lithium ion beams provide compelling diagnostic opportunities. Specifically, these FIBs are uniquely suited for studying lithium ion interactions in energy storage materials. As a result, it is of great interest to continue the development of cold-atom Li-ion sources to realize their full potential.

In this work, we report a Li^+^ MOTIS with a peak brightness of (1.2 ± 0.2) × 10^5^ Am^−2^ sr^−1^eV^−1^ and, in high-current mode, a maximum ion current exceeding 1 nA. These represent ≈ 20-fold improvements in beam brightness and ion current over the previous cold-atom Li^+^ source.^24^ A higher load rate is achieved by using larger MOT beams, which increase capture velocity, and by loading with a 2D MOT instead of a Zeeman slower, which provides an efficient conversion of hot, thermal atoms into a slow Li beam. Photoionization is achieved in a two-step process from the ground state rather than directly from the excited state of the cooling transition. Separating the photoionization pathway from the laser cooling cycle gives improved control over the geometry of the ionization region, which is defined by the intersection of two focused laser beams.^18,28^ Using an independent ionization pathway also decouples the ionization rate from the excited state fraction, and therefore from the MOT temperature.^29^ Finally, using a resonant first ionization stage reduces the required ultraviolet (UV) laser power by several orders of magnitude, shifting the burden of non-resonant ionization—which requires large power—to a more easily-amplified infrared (IR) laser.

We characterize the source by measuring the total current and the current density of the ionized beam and the temperature of the neutral gas. These experimental quantities allow us to determine a normalized brightness, which we then derate using a calculation of Coulomb interactions. We use the measured properties to predict the features, performance, and limitations of a FIB based on this source. By studying this system in detail, we provide in-depth understanding of MOTIS behavior and inform future MOTIS designs.

## II. DESIGN

**FIG. 1.**
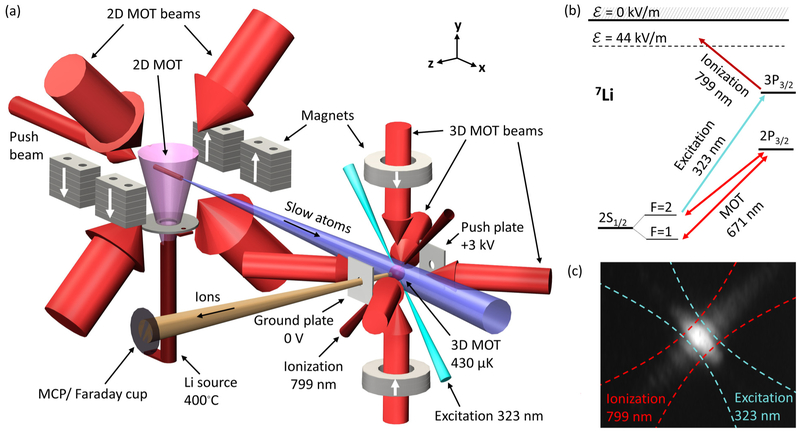
(a) Schematic of Li^+^ ion source. A slow beam of neutral ^7^Li is created using a 2D MOT loaded from an effusive thermal source. The atomic beam is captured in a 3D MOT and cooled to a temperature of ≈ 430 *μ*K. Two laser fields (excitation and ionization) are applied to ionize a small volume of the MOT. Plate electrodes accelerate the ions to form a beam, which is characterized using an MCP and a Faraday cup. White arrows indicate the magnetization direction of the magnets. (b) Level diagram of ^7^Li shows the MOT cooling transitions (trap and repump) as well as the excitation-ionization pathway. The electric field (*ε*) used for ion extraction lowers the ionization threshold, reducing the photon energy required in the ionization beam. Laser parameters are reported in Table I. (c) Example MCP image shows a representative cross section of the ion beam with overlaid beam envelopes showing the orientation of the crossed ionization lasers.

The apparatus for generating the Li^+^ ion beam comprises four principal parts: an effusive thermal oven for generating Li vapor, a 2D MOT which captures and redirects the low- velocity tail of the thermal vapor into a slow atomic beam, a 3D MOT to trap the atoms and cool them to low temperature, and a set of lasers and electrodes for ionization and beam formation. These components are illustrated schematically in Fig. 1(a). Not pictured in this image is the stainless steel vacuum chamber enclosing the apparatus, which is held at a base pressure of ≈ 10^−6^ Pa (≈ 10^−8^ Torr). The properties of the MOT are established using optical imaging of the trapped vapor and time-of-flight analysis to determine the temperature. The ion beam is imaged using a micro-channel plate (MCP), and its current is measured using a Faraday cup.

The effusive lithium source is housed in a stainless steel tube (height ≈ 5 cm, diameter ≈ 1 cm) mounted approximately 4 cm below the atomic beam axis inside a vacuum chamber. Immediately before pumping the chamber down to its base pressure, the tube is loaded with approximately 500 mg of metallic lithium in pellet form. By passing a DC current (≈ 30 A) through the steel tube, the lithium is heated to ≈ 400 °C, causing it to evaporate toward the 2D MOT. A K-type thermocouple is spot-welded to the tube to monitor temperature and enable closed-loop temperature control. A stainless steel aperture restricts line-of-sight between the source and the 2D MOT viewports, protecting them from Li deposition. This aperture allows the source to be placed much closer to the 2D MOT than would otherwise be possible, maximizing atom flux. No differential pumping is required between the Li source and the rest of the experiment, though the base pressure rises to a uniform ≈ 10^−5^ Pa (≈ 10^−7^ Torr) when the source is turned on.

Li atoms from the heated source are slowed and trapped in two dimensions by the 2D MOT and ejected by a push beam, forming a slow-moving atomic beam along the x-axis. This system closely resembles that described in Ref. 30, to which the reader is referred for further details. The 2D MOT is formed by four banks of Nd_2_Fe_14_B permanent magnets and two sets of circularly polarized, counter-propagating beams tuned to the 2S_1/2_−2P_3/2_ optical transition [670.96 nm; see Fig. 1(b) and Table I]. To avoid optical pumping into either 2S_1/2_ hyperfine state, the MOT beams contain two frequency components, referred to as trap (from state F = 2) and repump (from F = 1). Beam parameters and detunings for all MOT beams are reported in Table I. The 2D MOT magnets are oriented so as to produce a radial quadrupole field (gradient ≈ 0.5 T m^−1^) around the x-axis. The push beam is tuned to the trap transition and pushes the atoms in the +**x** direction, producing a collimated atom beam moving at ≈ 30 m/s.

The slow atoms propagate ≈ 24 cm before being recaptured in a 3D MOT formed by two stacks of Nd_2_Fe_14_B ring magnets and three sets of counter-propagating MOT lasers. The axially magnetized rings are oriented facing each other, producing a 3D quadrupole field with gradient ≈ 0.5 Tm^−1^ (≈ 0.25Tm^−1^) along the y- (x-, z-) axis. Because Li from the oven makes a 90° turn at the 2D MOT, the only atoms arriving at the 3D MOT are those that have already been trapped and cooled.

The 3D MOT is observed using two CMOS digital cameras and a Si photodiode. We determine the size of the trapped cloud through direct imaging of the fluorescencing atoms. By turning off loading from the 2D MOT and observing the decay of this fluorescence, we obtain the 3D MOT lifetime. The temperature is determined using a time-of-flight measurement, by turning off the 3D MOT beams and observing the expansion of the lithium cloud after a range of time intervals. The rate of cloud expansion in the absence of a trapping force yields a measurement of the velocity spread of the ensemble. The permanent magnetic field complicates this measurement slightly by slowing the low-field-seeking atoms—an effect we take into account in our analysis. We estimate the load rate by ionizing a fraction of the MOT and measuring the corresponding change in steady state fluorescence. We observe that MOT fluorescence, *F*, depends linearly on ion current, *I*. Explicitly, *F*(*I*) = *F*_0_ - *βI*, where *F*_0_ is the fluorescence of the undisturbed MOT and *β* is a proportionality constant. Assuming this linear relationship holds, the maximum current available in the MOT, which corresponds to the load rate, is *F*_0_/*β*. Depending on the laser power settings, we observe load rates between 10^8^ and 10^10^ s^−1^, with temperatures between 400 *μ*K and 3 mK. In general, higher-load-rate MOTs have higher temperatures. For the MOT used to collect the data in Figs. 3–6, we observe a peak density of ≈ 2.3 × 10^17^m^−3^, a load rate of ≈ 4.9×10^8^s^−1^, a lifetime (without ionization) of ≈ 31 ms, and a temperature of (430 ± 50) *μ*K.

At the center of the 3D MOT, we produce ions using a two-photon process. First, a UV beam at 323.36 nm, focused to a 1/e^2^ radius of ≈ 6.7 *μ*m, excites atoms to the 3P_3_/_2_ state [Figs. 1(b) and 1(c)]. This transition is resonant, and it requires relatively little power—below 1 *μ*W. The excited atoms are then ionized by an IR laser at ≈ 799 nm, which is focused to a 1/e^2^ radius of ≈ 9.2 *μ*m and oriented to cross the UV beam at a right angle. Being non-resonant, the optimal IR beam power is high—over 400 mW. In order to minimize heating of the beam during ionization, the wavelength of the second ionization stage is chosen to be below threshold for zero-field ionization, but above threshold in the presence of the extraction field (approximately 44 kV/m, as determined by a numerical simulation of the acceleration plates and the surrounding chamber). Past work has demonstrated that an ion beam formed by near-threshold ionization retains the temperature of the neutral ensemble.^32^ Ions accelerate in the +**z** direction along the electric field between a +3 kV push plate and a grounded electrode [Fig. 1(a)]. They travel ≈ 38 cm to either an MCP or a Faraday cup for characterization. We note that the extraction optics in this experiment are for diagnostic purposes and would be altered for use in a FIB.

Coherent light for this experiment is generated using three separate systems and stabilized using two Li spectroscopy cells. Light for the MOTs is produced in a commercial Ti: Sapphire laser system, tuned to 670.96 nm and stabilized using modulation transfer spectroscopy in a ≈ 10 cm vapor cell at 350 °C.^33-35^ UV light (323 nm) for the first ionization stage (excitation) is obtained from a commercial system combining a Ti:Sapphire tunable laser at 825 nm with its own 532 nm pump light in a non-linear frequency mixing cavity. The UV light is stabilized to a 2S_1/2_-3P_3/2_ transition using modulation transfer spectroscopy in a ≈ 70 cm heat pipe modeled on one described in Ref. 36. Because some field- and polarization-dependent spectroscopic structure is observed in the excitation rate at the MOT, we optimize the UV beam empirically by using a quarter wave plate to adjust the polarization and shifting the UV frequency to maximize ion current. The optimal frequency for this setup is approximately 10 MHz below the transition’s zero-field center-of-gravity. The second stage of ionization is carried out using up to 500 mW of 799 nm light from an extended cavity diode laser coupled with a homebuilt tapered amplifier. This stage of ionization is a broad transition that does not require active frequency stabilization.

**FIG. 2.**
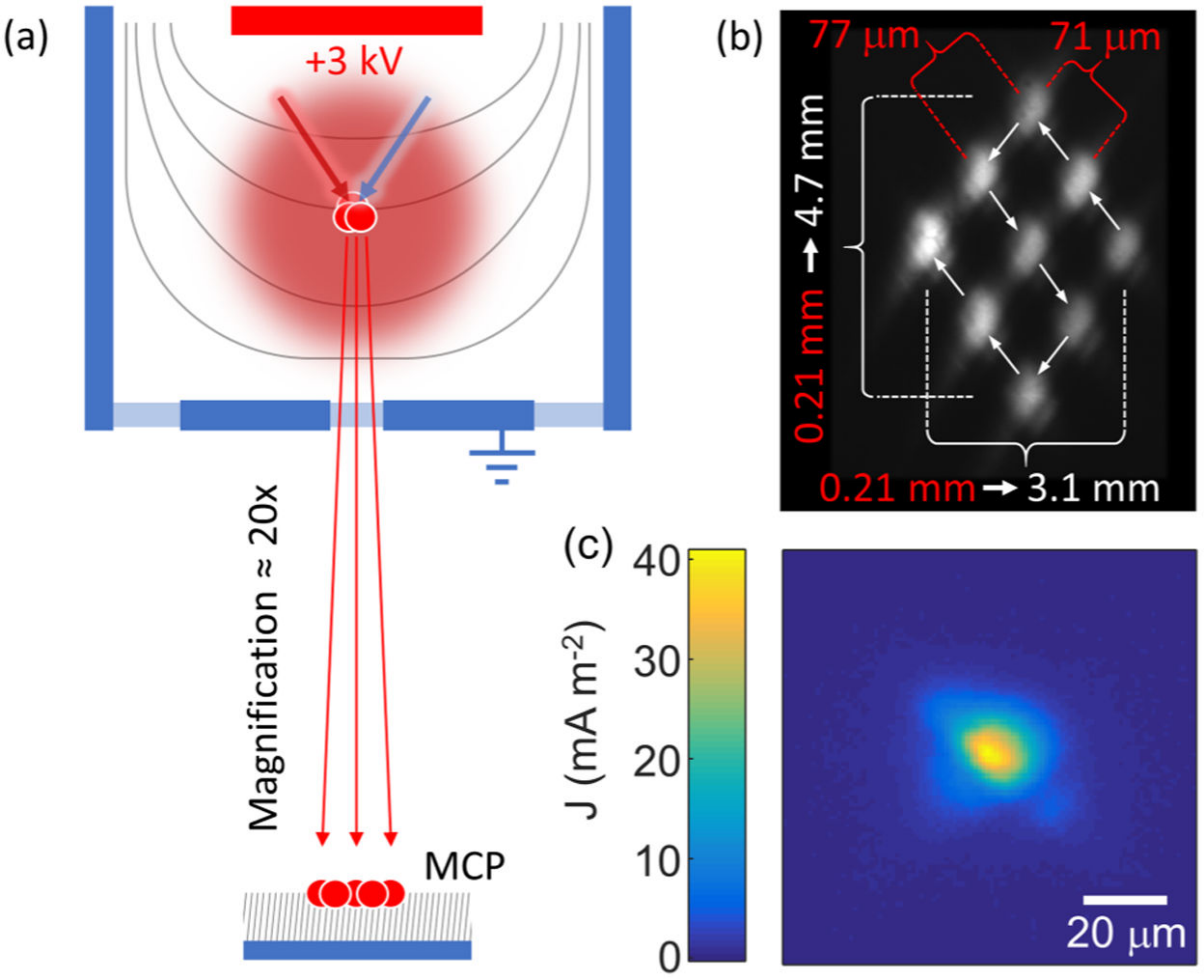
(a) Schematic of the extraction optics used to accelerate ions into a beam. A positively biased plate electrode pushes ions toward a grounded plate with an aperture at its center. Equipotential lines (shown in gray) are curved due to the grounded walls of the vacuum chamber. The result is a magnified projection of the ion source onto the MCP. (b) Composite image showing a 3 × 3 grid of beam profiles obtained by regular translations of the ionization lasers. The UV and IR beam waists are shifted by known increments of 77 ± 9 *μ*m and 71 ± 9 *μ*m, respectively. The resulting diagonal grid spans 210 ± 20 *μ*m in each direction. At the MCP, this grid has measured dimensions of 4.7 ± 0.3 mm and 3.1 ± 0.2 mm, yielding vertical and horizontal magnifications of 22 ± 2 and 15 ± 2, respectively. (c) Representative image of the ion source, where the magnification factors and the total current have been used to establish an undistorted length scale and a calibrated current density map. The excitation and ionization laser powers used for this image were approximately 70 nW and 425 mW, respectively.

We image the ion beam cross section using an MCP fitted with a phosphor screen. A digital camera records the phosphor screen fluorescence for analysis. In addition to imaging with the MCP, we measure the ion current directly using a Faraday cup. A linear translator allows us to move the cup into and out of the beamline, toggling between imaging and current measurements. The cross section of the beam at any point along its path provides a useful measure of the spatial distribution at the source, due to the high degree of collimation. However—because of the geometry of the vacuum chamber—the equipotential lines between the push plate and the drift tube are not parallel, but curved [Fig. 2(a)]. This curved field forms a divergent electrostatic lens, resulting in a magnified projection of the beam onto the MCP. We experimentally determine the mapping between the source and the projected image by translating the ionization beams in known distance increments and measuring the corresponding changes in the phosphor screen image [Fig. 2(b)]. We find that the magnification is astigmatic (due to different distances of the chamber walls), with horizontal and vertical magnification factors of M_x_ = 15 ± 2 and M_y_= 22 ± 2, respectively. No significant deviation from these numbers is observed over the relevant field of view. Numerical simulations of this system corroborate the observed magnification and confirm that millikelvin-scale ion temperatures contribute negligibly to image broadening. MCP images of the ion beam profiles are converted to current density maps by equating the total counts measured to the total ion beam current [Fig. 2(c)].

## III. RESULTS AND DISCUSSION

Our measurements of total beam current, current density at the source, and temperature provide a comprehensive characterization of the Li cold atom ion source, allowing comparison to other systems and prediction of focusing performance in a typical FIB column. We discuss the measured quantities in terms of a simple model, which helps us understand the regimes of operation and the limiting factors involved.

**FIG. 3.**
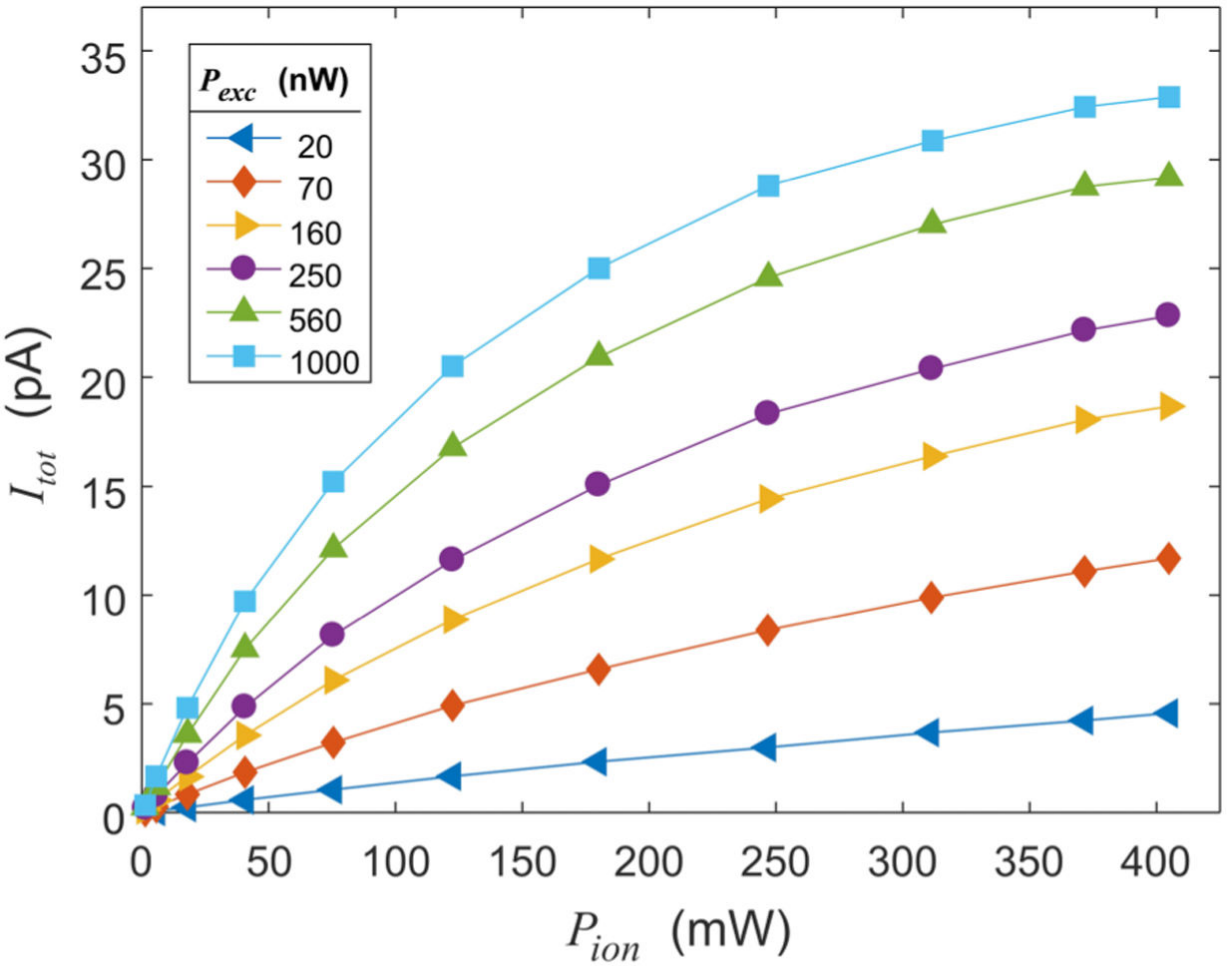
Total ion current (*I*_tot_) as a function of power in the IR ionization beam (*P*_ion_) for a range of UV excitation powers (*P*_exc_).

While total ion current increases monotonically with power in the excitation and ionization lasers, limiting factors play an important role. Figure 3 shows measurements of the total ion current (*I*_tot_) as a function of ionization power (*P*_ion_) for a range of excitation powers (*P*_exc_). The behavior in Fig. 3 can be understood as follows. When one laser scattering rate is significantly higher than the other, the low-power transition becomes a rate-limiting step, reducing the effect of the stronger laser. In this regime—referred to as the rate limit—the total current is low, limited by the two-photon transition rate. When both laser scattering rates are high, the current is limited by local depletion of the MOT density. In this regime—called the transport limit—the atoms are ionized faster than they can be replenished by transport into the ionization region.

**FIG. 4.**
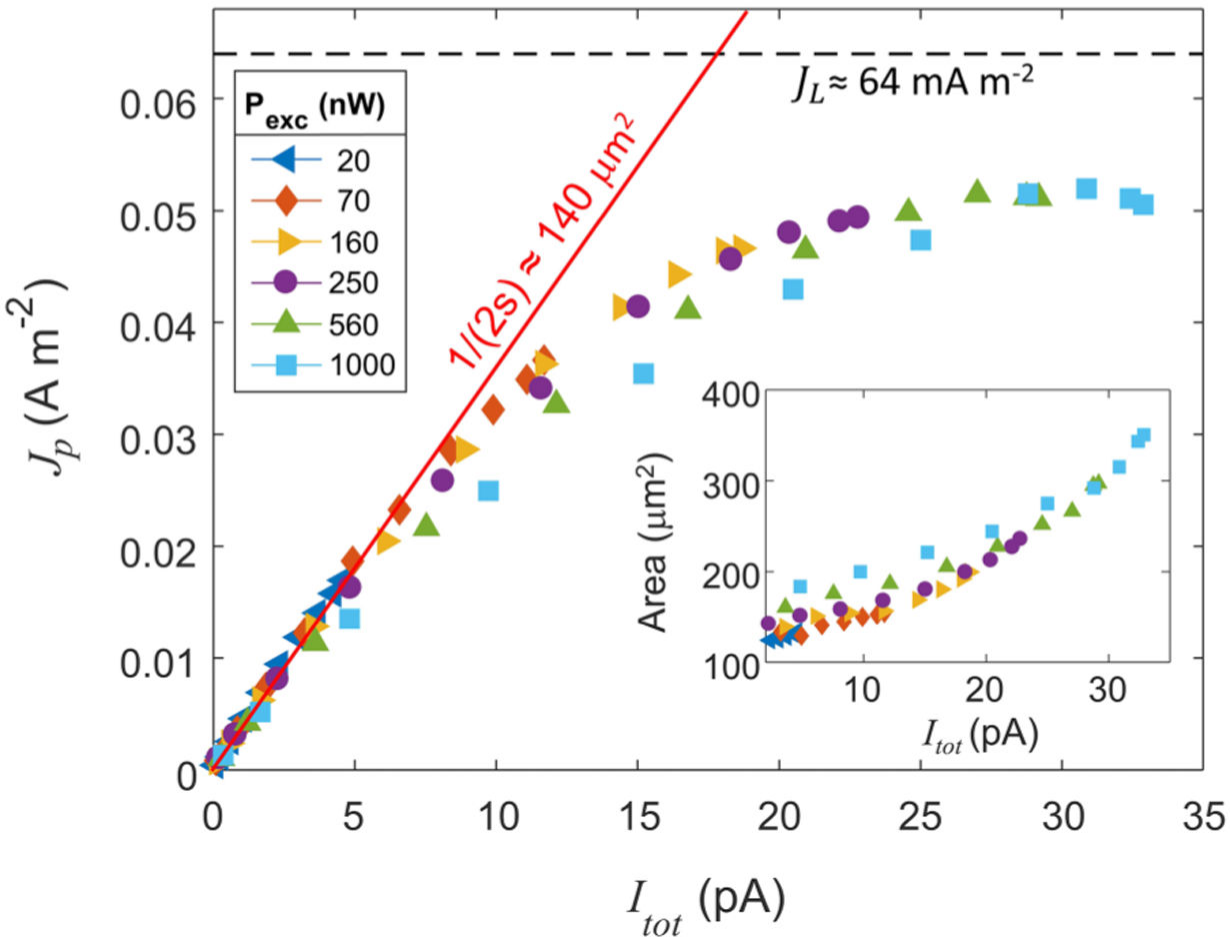
Peak current density (*J*_*p*_) as a function of total current (*I*_tot_) for a range of UV excitation powers (*P*_exc_). At low currents, the system is in the rate limit and *J_p_* ∝ *I*_tot_. The slope (s) of a linear fit to the data in this regime (red line) corresponds to source area of ≈ 140 *μ*m^2^. As the current increases, the size of the ionization region changes due to local MOT depletion and to saturation of the excitation transition. This causes the curves to fall below their initial slope. At high currents, the curves approach the transport limit, *J*_*L*_ (black dashed line). (Inset) Source area [calculated as the total cross-sectional area satisfying *J*(*x, y*) > *J_p_*/2] is plotted as a function of *I*_tot_ and shows how the size of the ionization volume depends on both UV power and total current.

In order to further explore the behavior of the source in the context of the two operating limits, we examine the relationship between peak current density (J_p_) and total current. For this analysis, we model the radial 2D current density as a Gaussian: J(*r*) = exp[−*r*^2^/*σ*^2^)]*I*_tot_/(2*πσ*^2^). The peak current density is then *I*_tot_/(2πσ^2^). In order for the plot of *J*_*p*_ vs *I*_tot_ to be linear, the cross-sectional area of the ionization region (i.e., πσ^2^) must remain constant. Deviations from linearity therefore indicate either a change in source size, a deviation from the Gaussian distribution, or both. Figure 4 and its inset show the clear connection between peak current density and source size. In the plot of *J*_*p*_ vs *I*_tot_, curves from different excitation laser powers all follow a similar arc, which grows linearly before leveling off at high current. The linear-growth regime in this figure corresponds to the rate limit, reflecting the fact that the size of the ionization region does not change with current. The linear relationship holds for a wide range of laser power distributions, provided the total current remains small. One notable exception, which we will address shortly, is when UV saturation effects broaden the waist of the excitation beam at high power. At high current, which corresponds to the transport limited regime, the local ionization rate approaches a fundamental limit.

Power in the UV excitation beam affects source size through saturation of the 2S_1_/_2_-3P_3_/_2_ excitation. Saturation intensity for this transition—nominally 0.65 mW/cm^2^—changes depending on the IR power, which depletes the excited state population through ionization. Nevertheless, the UV beam intensity in this experiment can exceed the nominal saturation threshold by a factor of 10^3^ or more. When the UV transition saturates, the excitation rate plateaus at the center of the beam. The current gained by increasing power therefore comes from the wings of the Gaussian intensity distribution. The inset of Fig. 4 shows that source size changes with excitation laser power, even when the total current remains fixed. This effect is apparent in the main plot of Fig. 4, where the peak current density at fixed current is smaller for high excitation powers.

In the transport limit, atomic flux into the ionization volume limits the maximum current density that can be generated in the source. This restriction is set by the density and velocity of atoms in the magneto-optically trapped gas.^1^ If atoms enter the ionization region at a rate determined entirely by their thermal velocity, $v=\sqrt{8k_{B}T∕(\pi m)}$, an upper limit on the steady state current density is *J*_L_ = 3*en_p_v*/2, where *n*_*p*_ is the peak density of the undisturbed MOT and *e* is the electron charge. Using the measured MOT properties listed above, we calculate *J*_L_ ≈ 64 mA m^−-2^. This limit, which is independent of source size, is shown as a black dashed line in Fig. 4. When the center of the ionization region is empty, the only way to increase current is by increasing the ionization volume, with flux increasing in proportion to the region’s surface area. In our system, this happens as laser intensity rises, increasing the ionization rate at the edges of the beams. In the high current regime of Fig. 4, we see that the current density saturates as the transport limit takes effect. Further exploration of this regime—including a theoretical description of MOT density and transport characteristics for specific laser power settings—is the subject of ongoing research.

In order to predict how our source will focus in a typical FIB column, we calculate brightness using MOT temperature and current density [Eq. (1)]. The assumption in using this formula, however, is that the temperature of the ions is the same as that of the cold atoms. While this can be true for near-threshold ionization at low currents and/or high extraction fields, it has generally been found that inter-ion Coulomb forces can cause a significant increase in beam temperature as the ions propagate through the system.^37^ To provide a more realistic value for the source brightness, we divide the brightness calculated in Eq. (1) by a Coulomb factor, *η_c_*, which is derived from Monte Carlo simulations following the procedure outlined in Ref. 37. These simulations yield the increased emittance *ε_final_* after a propagation distance of 7 mm, a distance beyond which the transverse temperature remains approximately fixed. The Coulomb factor is then calculated as $\eta_{c}=\varepsilon_{\text{final}}^{2}∕\varepsilon_{\text{initial}}^{2}$. We use a constant extraction field of 85.1 kV/m, chosen to keep the beam energy spread below 1eV (full width at half maximum). Because *η_c_* will depend strongly on the specific extraction dynamics of future setups, we include it as an error-free theoretical parameter. It is worth noting that a larger extraction field would reduce the Coulomb factor, increasing brightness by up to a factor of 3, at the expense of larger energy spread.

**FIG. 5.**
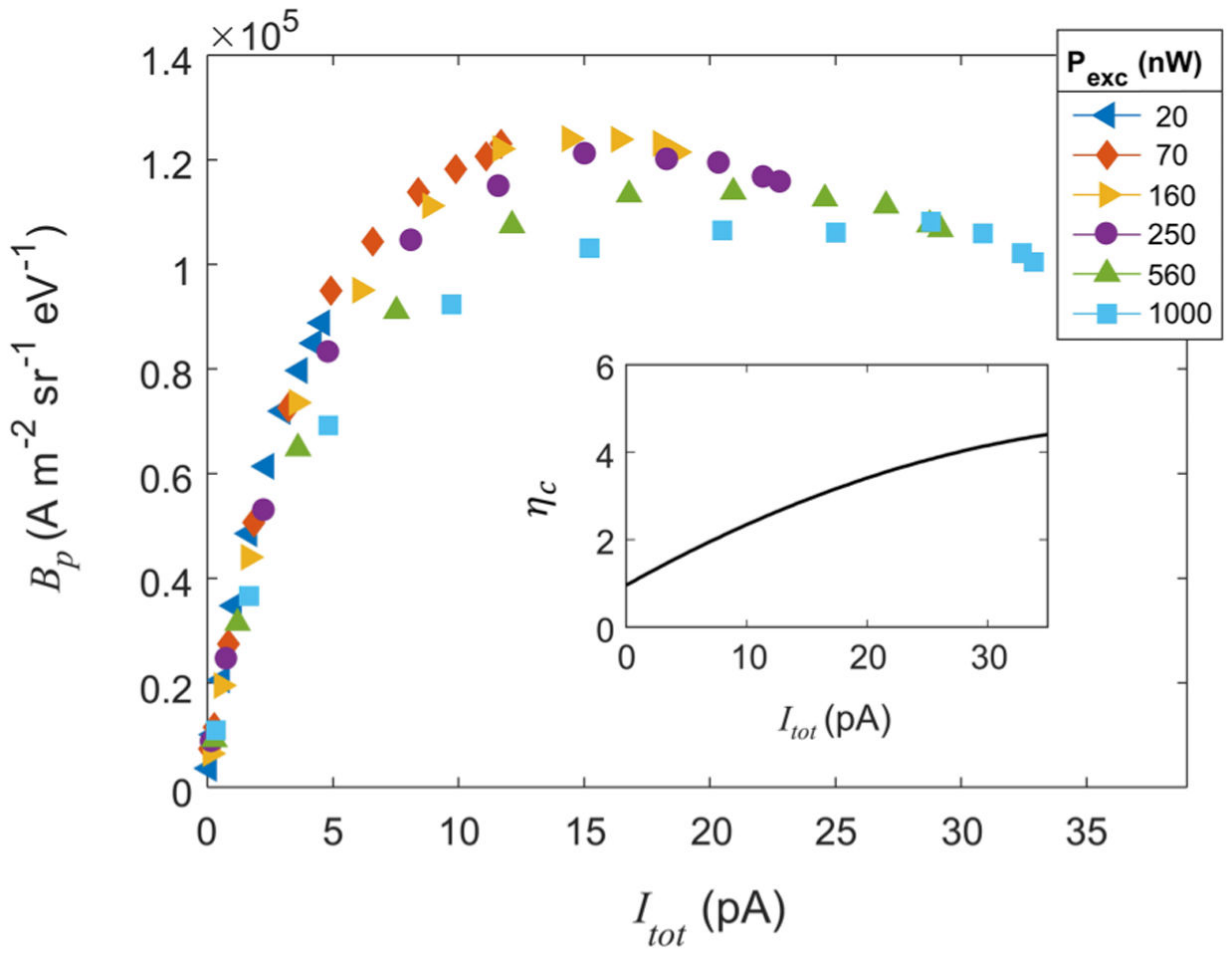
Peak normalized brightness (*B_p_*) as a function of total current (*I*_tot_) for a range of UV excitation powers (*P*_exc_). Optimal peak brightness occurs when the current density is large, but Coulomb effects are still small. *P*_exc_ and *P*_ion_ for the maximum observed brightness are 160 nW and 250 mW, respectively. (Inset) Coulomb factor (*η_c_*) as a function of total current.

We calculate the peak brightness, derating for Coulomb interactions, as *B_p_* = *J_p_*/(*πK_B_Tη_c_*). The relationship of this quantity to the total current is shaped by the rate and transport limits, as well as by Coulomb effects at high current. The maximum peak derated brightness observed for this source is (1.2 ± 0.2) × 10^5^ Am^−2^ sr^−1^ eV^−1^, obtained using *P_exc_* and *P_ion_* of approximately 160 nW and 250 mW, respectively. At the optimal operating current of (14.4 ± 0.7) pA, *η_c_* ≈ 3. Figure 5 shows peak normalized brightness as a function of total current, along with the Coulomb factor used to calculate it (inset). As in the case of peak current density, peak brightness at low currents is limited by the two-photon ionization rate. At high currents, both transport and Coulomb repulsion play important limiting roles.

**FIG. 6.**
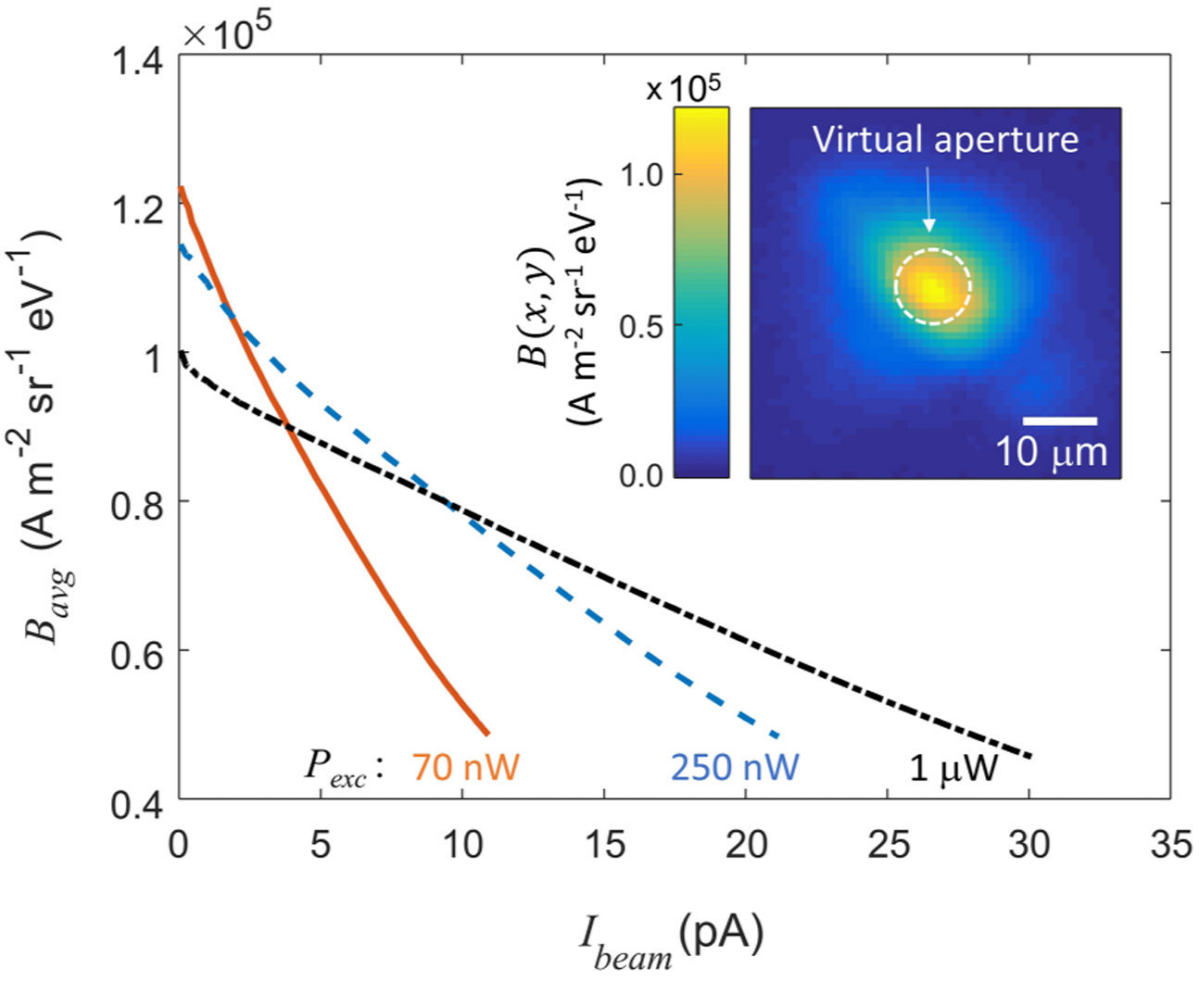
Average beam brightness (*B*_avg_) as a function of beam current (*I*_beam_), where the beam current is adjusted by varying the size of a virtual aperture. Coulomb heating is calculated based on *I*_tot_ and on the beam size before the aperture. For all three curves in this image, *P*_ion_ = 425 mW. (Inset) Representative brightness map for *P*_exc_ = 70 *μ*W and *P*_ion_ = 425 mW. A dotted line shows the boundary of a 10 *μ*m virtual aperture.

Peak normalized brightness offers an incomplete prediction of practical performance when the current density in a beam is not uniform, since a FIB uses more than just the brightest part of its source. We establish a more useful brightness metric by averaging *B*(*x, y*) = *J*(*x, y*)/(*πK_B_Tη_c_*) over a virtual aperture that transmits a circular region of the beam, centered on the brightest spot. We calculate the average brightness as a weighted sum over the portion of the beam passing through this aperture

(2) $$B_{\text{avg}}=\frac{\int \;B(x,y)J(x,y)dA}{\int J(x,y)dA},$$

where the integration is over the aperture area. As the size of the virtual aperture is varied, there is a trade-off between the usable current and the average brightness of the beam. Figure 6 shows *B*_avg_ as a function of beam current (*I*_beam_ ≡ ∫*J*(*x, y*)*dA*, where the integral is again over the aperture) for three different laser power configurations. We find that for low beam current (i.e., small beam apertures), the maximum average brightness is nearly the same as the peak brightness in Fig. 5. To obtain higher currents, larger virtual apertures can be used to sample a broader region of the source. In this case, the optimal laser power configuration might involve more power in the excitation beam, yielding lower peak brightness but better overall performance.

We can now estimate the spot size achievable with this source, assuming typical focusing.^17,38^ Referring to Fig. 6, we note that a beam with current (*I*_beam_) 1pA should have an average normalized brightness (*B*_avg_) of approximately 1.2 × 10^5^ Am^−2^ sr^−1^ eV^−1^. With a source size of 6 *μ*m and a local extraction field of 85.1 kV/m, we expect a longitudinal energy spread (ΔE) on the order of 1eV. Following the approach in Ref. 38, we estimate that 50% of the focused beam will fall on a spot with diameter

(3) $$d_{50}={\left[(d_{\text{br}}^{1.3}+d_{\text{sph}}^{1.3}{)}^{\frac{2}{1.3}}+d_{\text{cr}}^{2}\right]}^{1∕2},$$

where *d_br_* = [4I_beam_/(*B*_avg_E_0_π^2^*α*^2^)]^1/2^, *d*_sph_ = 2 ^−5/2^C_s_*α*^3^, and *d*_cr_ = 0.34C_*c*_ *α* Δ*E*/*E*_0_ are the respective size contributions from brightness, spherical aberration, and chromatic aberration, and *α* is the beam convergence angle. Selecting a beam energy of 30 keV and setting the spherical and chromatic aberration coefficients, *C_s_* and *C_c_*, to 100 mm and 30 mm, respectively, we find optimal focusing for a convergence angle just under 4 mrad. Under these conditions, *d*_50_ is approximately 4 nm.

For certain applications, it is desirable to operate a FIB with extremely large current, regardless of the brightness. We can achieve this by maximizing the ionization volume and the MOT load rate. To this end, we defocus the ionization lasers and set their powers to the highest values available in our system. We find that the optimal MOT parameters are very different in this mode. The trap detuning is larger—6Γ instead of 1Γ—and the 3D MOT laser powers are higher by a factor of nearly 5. The result is a much larger MOT, featuring a load rate of ≈ 8.6 × 10^9^ s^−1^. Under these conditions, we observe over 1 nA of current. Though the normalized brightness associated with this mode is fairly low—on the order of 10^3^ A m^−2^ sr^−1^ eV^−1^, derated for Coulomb interactions—it could prove useful for milling, large-area surface analysis, and high-flux implantation.

## IV. CONCLUSIONS

Novel approaches to loading and ionization have facilitated the construction of a cold-atom Li^+^ source that significantly outperforms previous lithium ion beams. By quantifying its performance over a range of conditions and comparing with simple models, we have established a robust understanding of the factors limiting this system. Beam brightness could be improved by increasing density and decreasing temperature in the MOT, though both metrics are within approximately a factor of two of their expected limits for the present configuration. More exotic traps, such as blue,^39^ gray,^40^ and multistage^41^ MOTs, could yield further gains, though potentially at the expense of transport and load rate. A combination of advanced cooling techniques and 2D-cooled designs could yield dramatic improvements at the cost of higher complexity. Further reductions in energy spread could be achieved using Rydberg ionization^42^—a topic which will be explored in future work.

This upgraded tool extends the possibilities for Li FIB microscopy, microanalysis, and implantation. It will enhance ongoing studies of lithium dynamics in battery electrode materials. Additional work is in progress to study the origins and theoretical features of the transport limit, which may give insight leading to further improvements.

## Figures and Tables

**TABLE I.** Properties of the cooling, trapping, and push beams used to collect the data reported in Figs. 3–6. All transitions take place between the 2S_1/2_ and the 2P_3/2_ states in ^7^Li. F and F′ represent the ground and excited hyperfine states, respectively, though the excited state hyperfine structure is not well resolved. In cases where several beams overlap (i.e., in the MOTs), the intensity represents the total power delivered by all beams. For reference, the linewidth of this transition^31^ is Γ/2π ≈ 5.9 MHz, and the saturation intensity is *I*_sat_ ≈ 2.54 mW cm^−2^.

Beam	Transition	Detuning	Intensity (mW cm^−2^)	Radius (1/e^2^)
2D MOT trap	F = 2 → F′=3	−6Γ	8.2	2.2 cm
2D MOT repump	F = 1 → F′=2	−4Γ	6.4	2.2 cm
3D MOT trap	F = 2 → F′=3	−1Γ	0.5	1.1 cm
3D MOT repump	F = 1 → F′=2	−4Γ	0.9	1.1 cm
Push	F = 2 → F′=3	−1Γ	11.2	1.4 mm
